# Invariant NKT Cell Response to Dengue Virus Infection in Human

**DOI:** 10.1371/journal.pntd.0002955

**Published:** 2014-06-19

**Authors:** Ponpan Matangkasombut, Wilawan Chan-in, Anunya Opasawaschai, Pisut Pongchaikul, Nattaya Tangthawornchaikul, Sirijitt Vasanawathana, Wannee Limpitikul, Prida Malasit, Thaneeya Duangchinda, Gavin Screaton, Juthathip Mongkolsapaya

**Affiliations:** 1 Department of Microbiology, Faculty of Science, Mahidol University, Bangkok, Thailand; 2 Systems Biology of Diseases Research Unit, Faculty of Science, Mahidol University, Bangkok, Thailand; 3 Center of Emerging and Neglected Infectious Diseases, Mahidol University, Bangkok, Thailand; 4 Medical Biotechnology Unit, National Center for Genetic Engineering and Biotechnology, National Science and Technology Development Agency, Pathumthani, Thailand; 5 Department of Pediatrics, Khon Kaen Hospital, Khon Kaen, Thailand; 6 Department of Pediatrics, Songkhla hospital, Songkhla, Thailand; 7 Dengue Hemorrhagic Fever Research Unit, Office for Research and Development, Faculty of Medicine, Siriraj Hospital, Mahidol University, Bangkok, Thailand; 8 Division of Immunology and Inflammation, Department of Medicine, Hammersmith campus, Imperial College London, London, United Kingdom; University of Pittsburgh, United States of America

## Abstract

**Background:**

Dengue viral infection is a global health threat without vaccine or specific treatment. The clinical outcome varies from asymptomatic, mild dengue fever (DF) to severe dengue hemorrhagic fever (DHF). While adaptive immune responses were found to be detrimental in the dengue pathogenesis, the roles of earlier innate events remain largely uninvestigated. Invariant natural killer T (iNKT) cells represent innate-like T cells that could dictate subsequent adaptive response but their role in human dengue virus infection is not known. We hypothesized that iNKT cells play a role in human dengue infection.

**Methods:**

Blood samples from a well-characterized cohort of children with DF, DHF, in comparison to non-dengue febrile illness (OFI) and healthy controls at various time points were studied. iNKT cells activation were analyzed by the expression of CD69 by flow cytometry. Their cytokine production was then analyzed after α-GalCer stimulation. Further, the CD1d expression on monocytes, and CD69 expression on conventional T cells were measured.

**Results:**

iNKT cells were activated during acute dengue infection. The level of iNKT cell activation associates with the disease severity. Furthermore, these iNKT cells had altered functional response to subsequent *ex vivo* stimulation with α-GalCer. Moreover, during acute dengue infection, monocytic CD1d expression was also upregulated and conventional T cells also became activated.

**Conclusion:**

iNKT cells might play an early and critical role in the pathogenesis of severe dengue viral infection in human. Targeting iNKT cells and CD1d serve as a potential therapeutic strategy for severe dengue infection in the future.

## Author Summary

Almost half of the world population is at risk of dengue viral infection. The disease severity varies from mild to a deadly form-which is caused mainly by host overt immune reaction. Earlier studies focused on the disease-causing roles of adaptive immune cells - cells that are highly specific but require time and signal from rapidly activating immune cells to become active. Invariant Natural Killer T (iNKT) cells are unique T cells that get activated rapidly and can control later adaptive response by secreting cytokines. They can be activated by lipid loaded on CD1d, an antigen presenting molecule. However, their role in human dengue infection was not known. Here, we studied iNKT cells from dengue infected children and found that they were activated. Importantly, the more severe the disease, the higher level of iNKT cells activation. Their cytokine patterns also differ from those of healthy donors. Moreover, together with iNKT cells activation, the level of CD1d was higher and T cells became active. Therefore, iNKT cells likely play a role in the pathogenesis of human dengue infection. New drugs targeting iNKT cells might help dampen the disease severity before the adaptive immune cells become too active.

## Introduction

Public health threat of dengue viral infection is expanding globally and most prominent in tropical and subtropical countries. It is the most significant mosquito- borne viral illness affecting mankind. An estimated 2.5 billion people live in the area at risk resulting in 50 to 390 million dengue infections per year [1]–[3]. Dengue infection causes significant morbidity, mortality and leads to hospitalization that consume vast amount of health care spending mostly in endemic areas where resource is scarce [1]–[3]. Currently, there is still no vaccine nor specific treatment, in part due to our incomplete understanding of the disease pathogenesis.

Dengue virus (DV) is a single stranded RNA virus in the *Flaviviridae* family. Four serotypes of dengue virus are circulating and cause human illness. The transmission from human to human requires *Aedes* mosquito vectors [4]. Once infected with DV, the clinical manifestation varies widely from asymptomatic infection, undifferentiated febrile illness to a more typical dengue fever (DF) characterized by fever associated with severe headache, myalgia, and bone pain, which are mild and self limited. A small percentage of patients develop a more severe, life threatening dengue hemorrhagic fever (DHF), which could result in dengue shock syndrome (DSS) [1]. The hallmarks of DHF/DSS are plasma leakage and hemorrhage that could lead to shock and death which invariably occur within 1–2 days after fever subsided [1], [5].

Both viral and host factors were shown to contribute to the severity of dengue infection [1], [6]. While protective immune response is required for viral clearance, the detrimental immune reaction was suggested to be the major cause of severe DHF/DSS [6]. Most previous investigations focused on the detrimental effects of cross-reactive adaptive immune responses known as antibody dependent enhancement [7]–[9] and T cell antigenic sin [10]. However, the study on innate and innate-like immune response in dengue infection that could initiate or even dictate the type of subsequence adaptive immune response is still very limited.

Invariant natural killer T (iNKT) cells represent a unique population of T cells with innate-like function. iNKT cells express invariant T cell receptor (iTCR) recognizing lipid antigens on CD1d, a non-polymorphic MHC class I –like molecule [11]. α-Galactosylceramide (α-GalCer) is a potent and specific iNKT cell antigen widely used for iNKT cell activation and identification [11]. A number of exogenous lipid antigens from microbes [12]–[15] and endogenous lipid antigens [16]–[19] were also shown to activate iNKT cells. Upon activation, iNKT cells can rapidly secrete a variety of preformed cytokines such as IFN-α, IL-4, IL-17A, IL-10 and crosstalk with conventional T cells, B cells, NK cells, macrophages and dendritic cells [11]. iNKT cells were shown to play a role in many disease processes including cancer, autoimmunity, asthma, and infections [11], [20], [21]. Their roles in infections were demonstrated for a variety of microorganisms including viruses [13]–[15], [22]–[25].

The critical roles of iNKT cells were demonstrated in several virus infections [24], [25] such as herpes simplex virus type 1 and 2 [26]–[28], respiratory syncytial virus [29], influenza A virus [30]–[32], hepatitis B virus [33], cytomegalovirus [34], [35], hepatitis C virus [36], and HIV-1 [37]. Moreover, patients with primary immune deficiency resulting in iNKT cell defect are more susceptible to severe varicella-zoster virus [38] and EBV infections [39]. Because of these evidence, iNKT cells activation by α- GalCer have been tested as vaccine adjuvant with promising results in animal models of hepatitis B virus [40], influenza A virus [41] and HIV infections [42], [43].

The potential role of iNKT cells in pathogenesis of DV infection has never been investigated in human. Two previous reports showed increased number and recruitment of T cells bearing NK cell markers to skin infected with DV in mouse [44] and marmoset models [45]. However, these reports did not use standard methods to identify iNKT cells, so it is unclear if these cells are truly iNKT cells. The only study of iNKT cells in DV infection was done in experimental murine model [46] and suggested a detrimental role of iNKT cells in severe form of DV infection. However, DV does not naturally infect mice and the use of mouse model to represent DV infection pathogenesis in human remains controversial [47]–[49]. Therefore, the study of iNKT cells in human patients is needed.

Here, we investigated the potential role of iNKT cells in DV infection in human by examining well-defined clinical samples from a cohort of DV infected children of varying disease severity and at different time points, in comparison to controls. We found that iNKT cells were activated in acute DV infection, and suggesting the involvement of iNKT cells in human severe DV infection. A better understanding of the role of iNKT cells in human DV infection will lead to a better understanding of the intricate control of complex immune responses and immunopathogenesis in dengue infection. Altogether, the advancement in our knowledge will enable the development of novel preventive and therapeutic approaches in the future.

## Methods

### Ethics statement

Blood samples were collected from healthy controls and 1–15 year-old patients admitted to Khon Kaen and Songkhla hospitals, Thailand, following written parental informed consent. This project was approved by the ethical committees of Khon Kaen hospital, Songkhla hospital and Mahidol University.

### Clinical samples

Peripheral blood mononuclear cells (PBMC) were separated from whole blood by Ficoll-Hypaque density gradient centrifugation and kept in liquid nitrogen for subsequent study. Acute dengue infection was determined by dengue gene identification using reverse transcription PCR (RT-PCR) and dengue virus-specific IgM capture ELISA as previously described [50], [51]. Dengue disease severity was classified according to the World Health Organization criteria (1997) [5] into dengue fever (DF) and dengue hemorrhagic fever (DHF). Other non-dengue febrile illness (OFI) patients were defined as patients hospitalized with fever without the presence of dengue infection by both RT-PCR and dengue virus-specific IgM capture ELISA.

Blood samples from each patient were collected at different time points during the course of infection. The date were called in relation to the day fever subsided (day of defervescence, day 0) so that “day -1” is one day before the day of defervescence and “week 2” and “month 6” are 2 weeks and 6 months after the day of defervescence, respectively. Random PBMC samples from DF, DHF and OFI were used for analysis in this study.

Samples from 11 DF, 19 DHF, 11 OFI patients and 10 healthy controls were evaluated for percentage and phenotype of peripheral blood iNKT cells. The demographic and clinical characteristics, including age, gender, DV serotype, lowest white blood cells count (WBC), highest hematocrit (Hct), lowest platelet (Plt), highest serum aspartate transaminase (AST), alanine transaminase (ALT) enzyme, and lowest albumin of each patient with DF and DHF are shown in Table S1. A different set of patient samples were used to study the function of iNKT cells by *ex vivo* stimulation due to limited number of PBMC available from each patient. Fourteen DF, 12 DHF, 11 OFI patients and 10 healthy controls were used in these experiments. Table S2 shows demographic and clinical characteristics of the DHF and DF patients whose PBMC were used for functional analysis. All patients studied had secondary DV infection.

### iNKT cell and T cell phenotypic analysis

To analyze the number and phenotypes of iNKT cells and conventional T cells, cryopreserved PBMCs were thawed and rested in cold complete RPMI medium. Cell numbers and viability were estimated by trypan blue exclusion assay. More than 90% cell viability were observed in all samples. Cells were then washed, Fc blocked and stained with the following fluorescence-conjugated monoclonal antibodies; PEcy7-conjugated anti-human CD3 (BioLegend; CA), PE-conjugated PBS57-loaded CD1d tetramer or PE-conjugated unloaded CD1d tetramer (NIH tetramer facility, USA; PBS57 is an α-GalCer analog), FITC-conjugated anti-human CD4 (BD Pharmingen), APCcy7-conjugated anti-human CD8 (BioLegend; CA), and PerCP- conjugated anti-human CD69 (or isotype control) (BD Biosciences) according to the manufacturer's protocol. Cells were then analyzed by flow cytometry.

### Cell culture, *ex vivo* stimulation and intracellular cytokine analysis

Cryopreserved PBMCs were thawed and cultured in the presence or absence of α-GalCer 100 ng/ml (KRN7000, Funakoshi, Tokyo, Japan) for 12 hours with brefeldin A (10 mg/ml; Sigma-Aldrich). Cells were then harvested and stained for the following surface markers and intracellular cytokines after permeabilization according to manufacturer's protocol: PEcy7 conjugated anti-human CD3 antibody (BioLegend; CA), PE conjugated PBS57-loaded CD1d tetramer (NIH, USA), PerCP conjugated anti-human CD69 antibody (BD Biosciences), FITC-conjugated anti-human IFN-γ (BD Pharmingen), APC-conjugated anti-human IL-4 (eBioscience) and Alexa700-conjugated anti-human IL-17A (BD Pharmingen) or isotype controls. Cells were then fixed with 1% formaldehyde in PBS and analyzed by flow cytometry.

### Monocyte CD1d expression

PBMC were stained with FITC-conjugated anti-human CD14 antibody (BD Pharmingen), and PE-conjugated anti-CD1d antibody (BD Pharmingen) or isotype control (BD Pharmingen). Cells were then fixed and acquired by flow cytometry. Monocytes were gated based on their characteristic appearance on FSC/SSC dotplot and upon CD14 expression. The level of CD1d expression was expressed as the difference in mean fluorescence intensity (DMFI) between CD1d staining and isotype control of each sample as baseline.

### Flow cytometry

Flow cytometry analysis was performed using BD FACS Canto or BD LSRII flow cytometer via FACS Diva version 4.1.1 software. FlowJo version 8.7 (Tree star) was used for data analysis.

### Statistical analysis

Data analysis was performed using GraphPad Prism 5.0 software and SPSS version 20. Mann-Whitney test was used for comparison of unpaired data. Wilcoxon signed rank test was used to compare paired data. Spearman's rho correlation test was used for correlation analysis of non-parametric data. P-value of <0.05 was considered as statistically significant difference.

## Results

### Demographic and clinical data

The age and gender of patients with DF and DHF in this study were not significantly different. The most common DV serotypes in both groups were DV serotype 1 (DV1), followed by DV2, DV3, and DV4, respectively. As expected, patients with DHF had significantly lower platelet count, serum albumin and higher liver transaminases when compared to those with DF (Table S1 and S2).

### The percentage of peripheral blood iNKT cells remain unchanged during the course of DV infection

**Figure 1:**
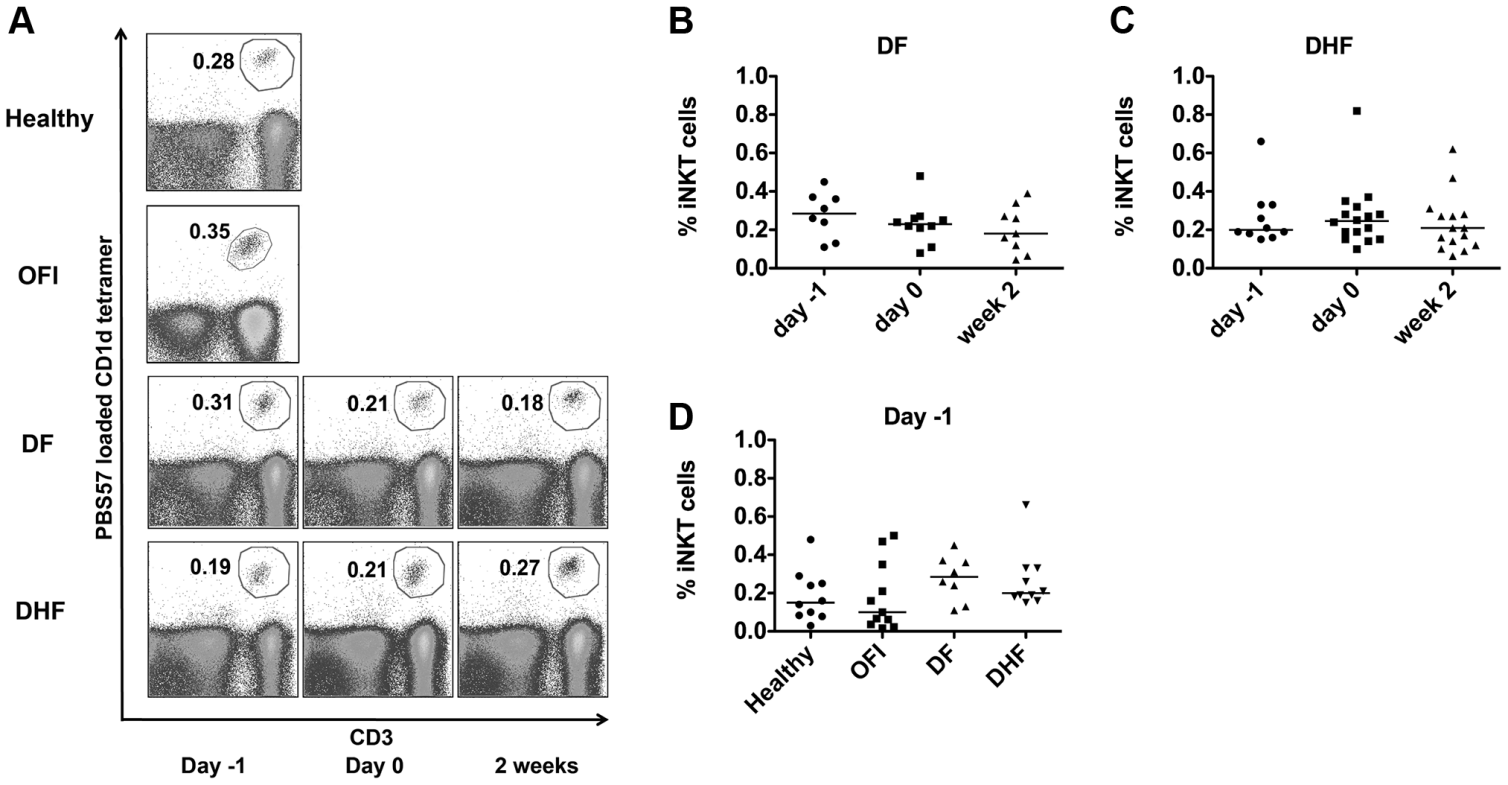
The frequency of peripheral blood iNKT cells during dengue infection with different disease severity. a) Representative dot plots show the percentage of iNKT cells within lymphocytes in healthy, other febrile illness (OFI), dengue fever (DF), and dengue hemorrhagic fever (DHF) at day -1, day 0 and day 2 weeks. b, c) Each dot represents percentage of iNKT cells of each patient in DF (b) and DHF (c) groups at 3 different time points, the line represents median of each group. d) The percentage of iNKT cells during febrile phase (day -1) of healthy, OFI, DF and DHF. Mann-Whitney test (b–d) was used for statistical comparison, p<0.05 was considered as statistically significant difference. No significant difference was found.

To examine whether the percentage of iNKT cells changes during the course of DV infection, PBMC from DF and DHF patients were evaluated at 3 different time points (day -1, day 0 and day 2 weeks) as previously described (Figure 1a). Lymphocytes were pregated based on their characteristic appearance on forward and side scattered dot plot. iNKT cells were then identified by the expression of both CD3 and PBS57- loaded CD1d tetramer (as compared to unloaded CD1d tetramer control) (Figure 1a, 2a). The percentage of iNKT cells within total lymphocytes were not significantly changed over the course of DV infection in both patients with DF (Figure 1b, Figure S1a) and DHF (Figure 1c, Figure S1b). When comparing iNKT cells from patients with various severities during febrile phase and controls, the percentage of iNKT cells were also not significantly different between groups of patients (Figure 1d). Absolute number of iNKT cells also showed similar results (Figure S1c-g).

**Figure 2:**
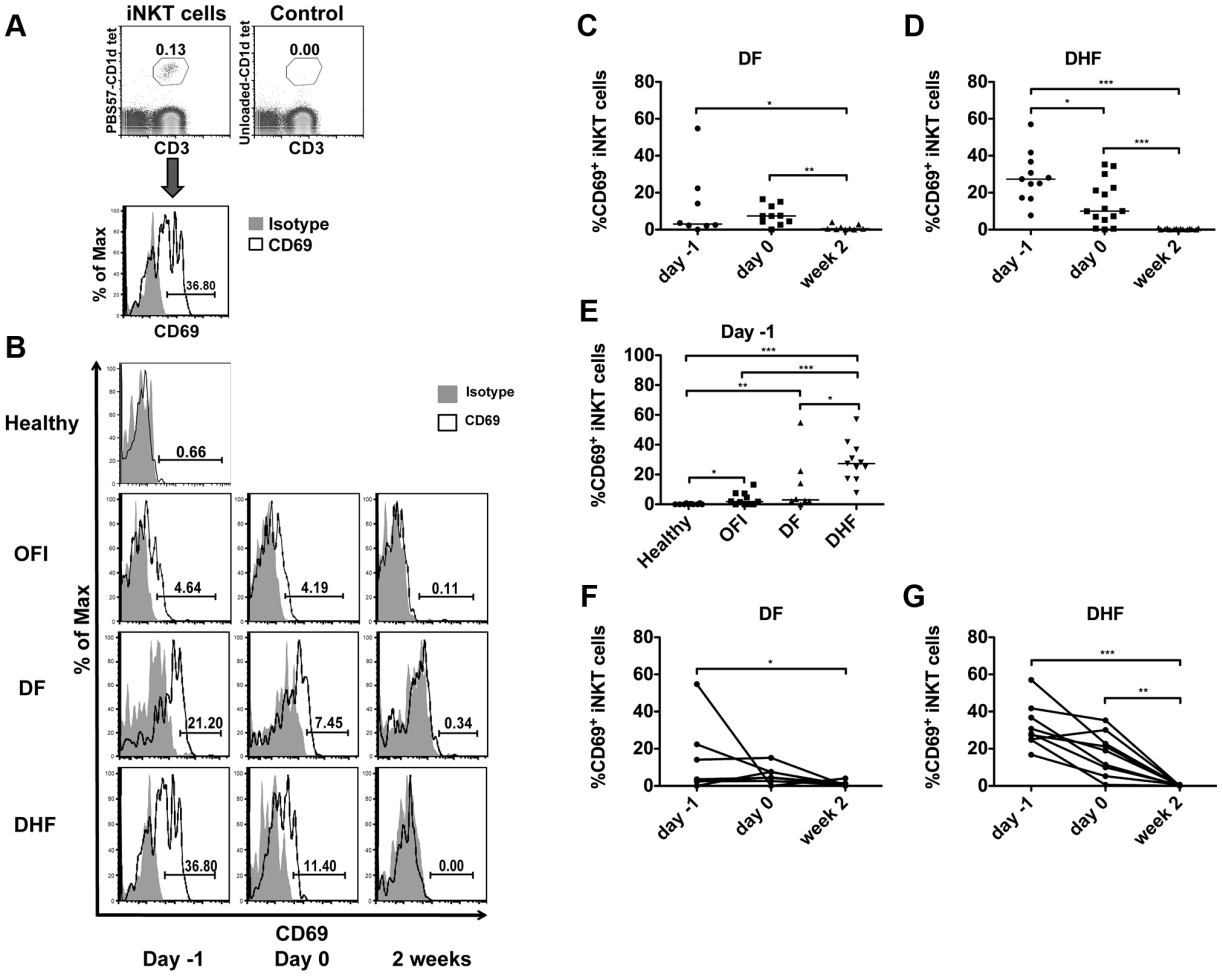
iNKT cells were activated during acute phase of dengue infection. a) Representative dot plot and histogram demonstrate gating strategy for iNKT cells and CD69 expression on iNKT cells. iNKT cells were gated, within lymphocytes population, on CD3^+^, PBS57-loaded CD1d tetramer^+^ (in comparison with unloaded CD1d tetramer control). CD69^+^ iNKT cells were gated in comparison to isotype control. b) Flow cytometry analysis of the expression of CD69 on iNKT cells in Healthy, OFI, DF, and DHF at day -1, day 0 and 2 weeks. Representative histograms show the expression of CD69 (black line) in comparison to isotype control (gray shade). c, d) Each dot represents percentage of CD69 positive iNKT cells of each patient in DF (c) and DHF (d) groups at 3 different time points, the line indicate median of each group. e) Dot plot summarized percentage of CD69 positive iNKT cells during febrile phase (day -1) of each group of patients in comparison to controls. f, g) Percentage of CD69 positive iNKT cells during the course of dengue infection, each line connects data from each patient at various time points. Mann-Whitney test (c–e), and Wilcoxon signed rank test (f–g) were used for statistical comparison, p<0.05 was considered as statistically significant difference (*p<0.05, **p<0.01, ***p<0.001).

### iNKT cells were activated during acute DV infection and the level of activation associated with dengue disease severity

To investigate whether iNKT cells are activated *in vivo* during human DV infection, peripheral blood iNKT cells from dengue-infected individuals with various disease severities were evaluated at different time points. Expression of CD69 in comparison to isotype control was used as an activation marker of iNKT cells (Figure 2a).

iNKT cells were activated during acute phase (day -1 and day 0) of dengue infection in both DF and DHF patients (Figure 2b). The percentage of CD69 positive iNKT cells was significantly higher during febrile phase (day -1) (median 3.02%; interquartile range 2.18–20.33%) and defervescence phase (day 0) (7.42%; 3.91–13.23%) compared to 2 weeks after fever subsided (0.50%; 0.13–2.08%) in patients with DF (p<0.05 and p<0.005 respectively) (Figure 2c). In patients with DHF, the percentage of CD69 positive iNKT cells was also higher during febrile phase (day -1) (27.40%; 17.20–36.80%) and defervescence phase (10.00%; 5.26–22.70%) when compared to 2 weeks after fever subsided (0.23%; 0.00–0.57%) (p<0.0001 and p = 0.0001, respectively) (Figure2d). Furthermore, when comparing iNKT cells from patients with various severities during febrile phase (day -1), patients with DHF have significantly higher percentage of activated iNKT cells (27.40%; 17.20–36.80%) when compared to DF (3.02%; 2.18–20.33%) (p = 0.0149) (Figure 2e), suggesting that the activation of iNKT cells associated with disease severity. Moreover, iNKT cells were more activated in dengue-infected patients (DF and DHF) than in OFI (1.77%; 0.00–7.33%) and healthy controls (0.00%; 0.00–0.51%) (Figure 2e). iNKT cell activation in OFI is higher than healthy controls (p<0.05), possibly due to the heterogeneous non-dengue infectious etiology of OFI group, some of which could also activate iNKT cells.

When cells from each patient were analyzed at 3 different time points, the data clearly showed that the percentage of activated iNKT cells were highest during febrile phase, continuously decreased over the course of infection and barely present by 2 weeks after fever subsided in both patients with DF (Figure 2f) and DHF (Figure 2g). No correlation between iNKT cell activation and DV viral load was observed (data not shown). Therefore, our results showed that iNKT cells were activated during acute dengue infection and the level of activation associated with dengue disease severity.

### Peripheral blood iNKT cells from acute DV infected patients had reduced α-GalCer mediated production of IFN-γ

**Figure 3:**
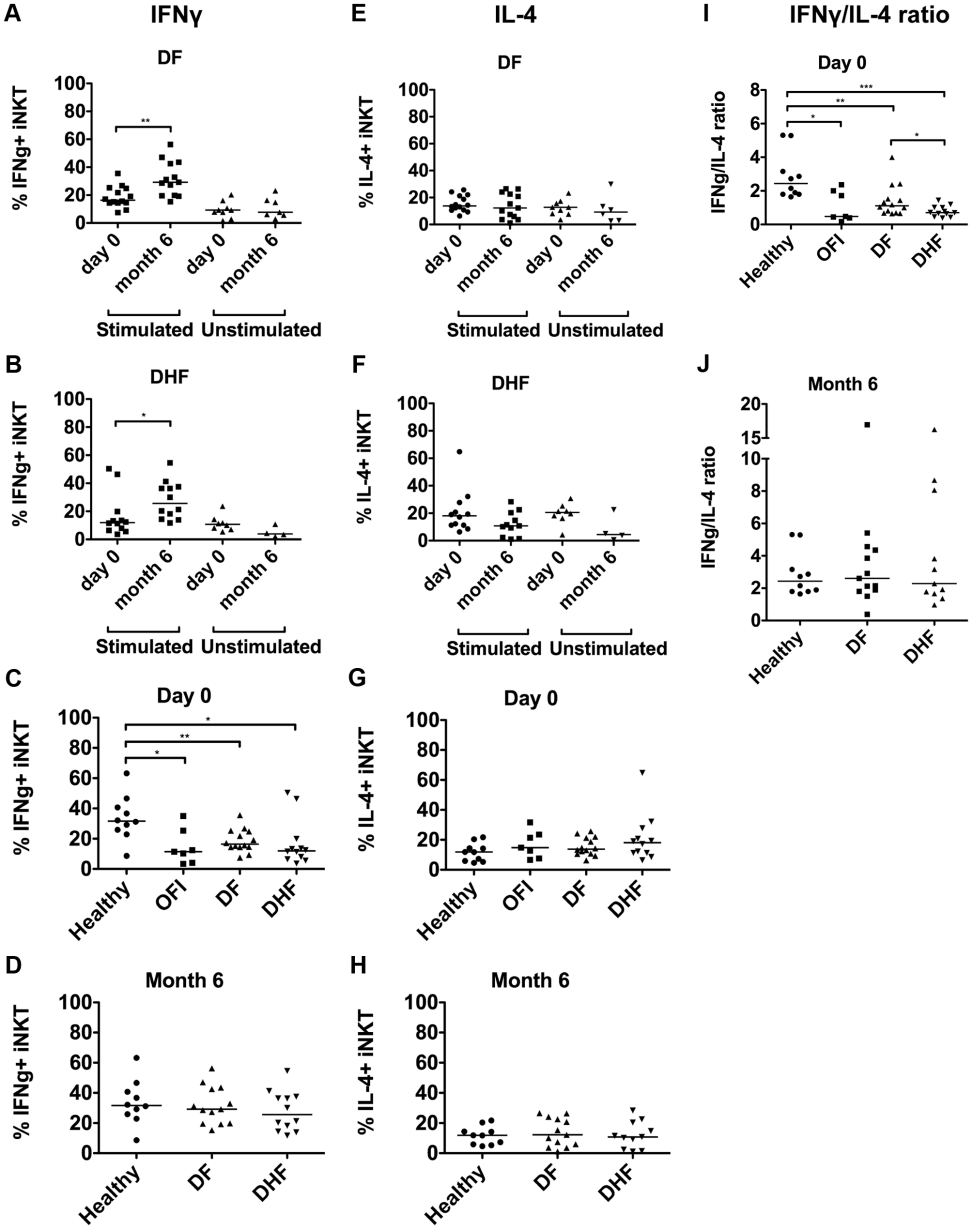
Cytokines production by iNKT cells from dengue infected patients with and without stimulation with α-GalCer. Each dot represents percentage of interferon gamma (IFN-γ)^+^ (a–b) and IL-4^+^ (e–f) iNKT cells from DF (a,e) and DHF (b,f) at day 0 and month 6, with (stimulated) and without (unstimulated) α-GalCer stimulation. At day 0 (c, g, i) and 6 months (d, h, j), percentage of IFN-γ^+^ iNKT cells (c,d), IL-4^+^ iNKT cells (g, h) and IFN-γ/IL-4 ratio (i, j) upon α-GalCer stimulation, comparing cells from healthy, OFI, DF and DHF groups. Mann-Whitney test, were used for statistical comparison, p<0.05 was considered as statistically significant difference (*p<0.05, **p<0.01, ***p<0.001).

To study function of the iNKT cells in DV infection, iNKT cells production of various cytokines (IFN-γ, IL-4 and IL-17) were analyzed by intracellular cytokine staining of gated iNKT cells in comparison to isotype controls. Without stimulation, a small amount of IFN-γ and IL-4 could be detected in iNKT cells from some of the acute dengue-infected patients (Figure 3a–b, 3e–f).

Previous reports demonstrated that iNKT cells could become anergic or hyporesponsive to α-GalCer stimulation if they were previously activated *in vivo* [52], [53]. To further examine the functional properties of the activated iNKT cells from acute DV infected patients, the PBMC were stimulated with α-GalCer *ex vivo* and intracellular cytokines were evaluated. PBMC from DF and DHF patients during acute dengue infection (day 0) were compared to those 6 months after infection, when the immunological effects of acute DV infection were assumed to return to baseline. The results were then compared with unstimulated condition and with PBMC from OFI and healthy subjects (Figure 3).

As expected, after α-GalCer stimulation, healthy iNKT cells produced large amount of IFN-γ (Figure 3c). In contrast, the capacity to produce IFN-γ of iNKT cells from acute DV infected and OFI patients was reduced (Figure 3c, Figure S2a). At day 0, after stimulation, the percentage of IFN-γ^+^ iNKT cells of patients with DF (16.35%; 14.40–24.93%) and DHF (11.95%; 6.77–18.30%) were lower than those of healthy control (32.1%; 25.90–40.70%) (p = 0.002 and p = 0.01, respectively) (Figure 3c). Therefore, the functional change of iNKT cells is not limited to dengue infection but also occur in other febrile illness, again, this could be due to the heterogeneous infectious etiology of OFI. No significant difference between DF and DHF was observed. The responsiveness to α-GalCer stimulation returned 6 months after fever subsided to the level similar to those of healthy control (Figure 3d). After α-GalCer stimulation, the percentage of IFN-γ^+^ iNKT cells from day 0 were significantly lower than those from 6 months after fever subsided in both patients with DF (day 0: 16.35%; 14.40–24.93% vs. month 6: 29.20%; 19.65–43.00%, p = 0.002) (Figure 3a) and DHF groups (day 0:11.95%; 6.77–18.30% vs. month 6: 25.65%; 15.35–37.23%, p = 0.01) (Figure 3b). No statistical significant difference was observed when IL-4^+^ iNKT cells were examined after α-GalCer stimulation (Figure 3e–h, Figure S2b).

Interestingly, the iNKT cells cytokine patterns in DF and DHF appears to be different. Acute DF patients have higher IFN-γ/IL-4 ratio compared to DHF (Figure 3i), suggesting that Th1-like response of iNKT cells in acute DV infection may associate with less disease severity.

IL-17A was not detectable in iNKT cells in all conditions with the current stimulation protocol (data not shown), perhaps because the kinetics of IL-17 is different from IL-4 and IFN-γ.

To further examine the kinetics of functional response of iNKT cells to α-GalCer, additional time points at day -1 and week 2 were studied (Figure S3 and S4). The responses at these 2 additional time points were not statistically significant different. However, iNKT cells from DHF at 2 weeks appeared to produce more IFN-γ than at day -1 but due to the limited number of sample, statistical test cannot be performed (Figure S4b). Altogether, the IFN-γ response to α-GalCer seems to reduce during acute DV infection and recover by month 6 after acute DV infection but the rate of recovery may differ between patients.

Taken together, these findings suggested that iNKT cells from acute dengue infected patients were previously activated *in vivo* and upon restimulation with α-GalCer *ex vivo*, they have reduced IFN-γ production. Importantly, skewing toward Th1-like cytokine pattern of iNKT cells during acute DV infection may associate with less clinical severity.

### Upregulation of CD1d expression on monocytes during acute DV infection

**Figure 4:**
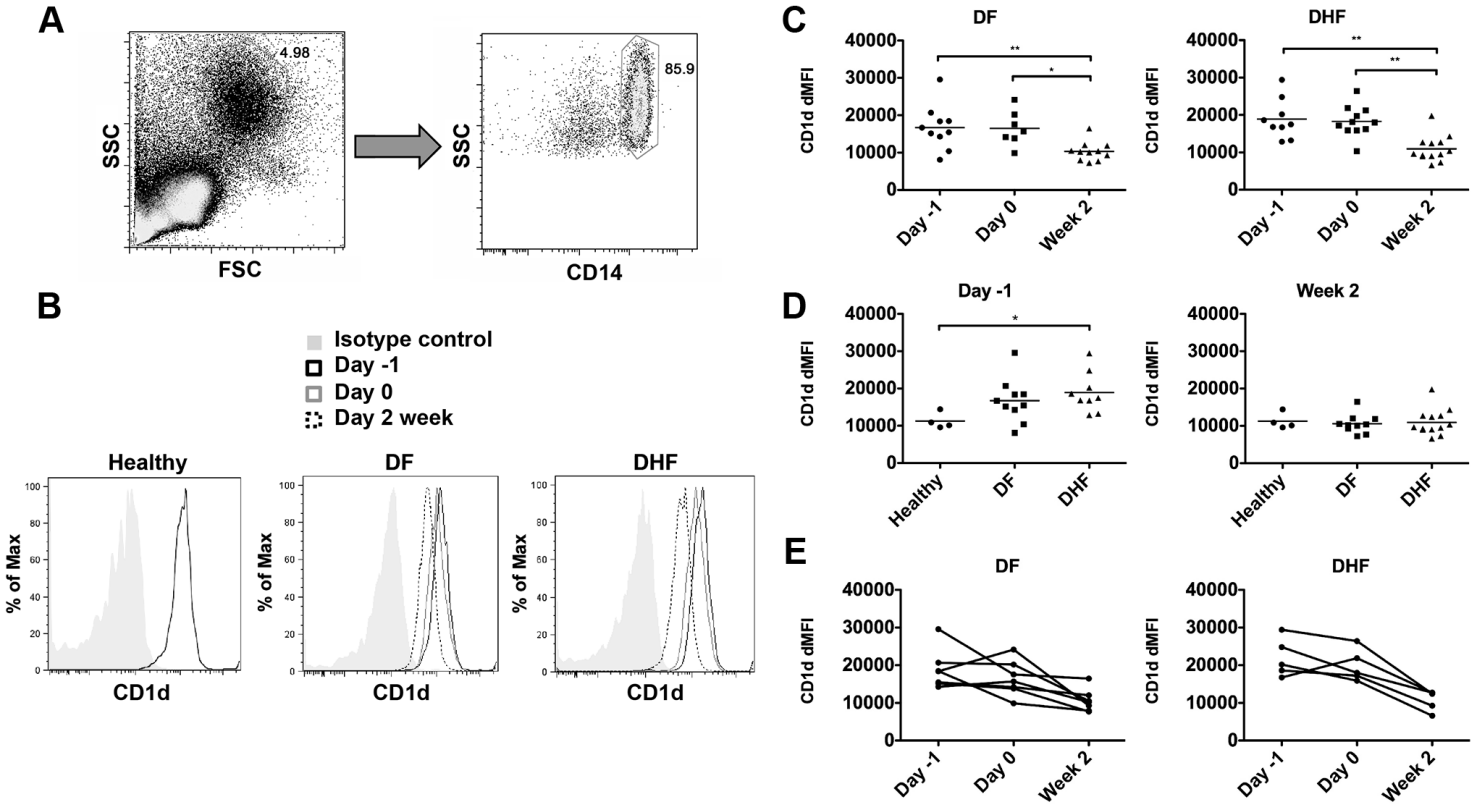
Upregulation of CD1d expression on monocytes during acute DV infection. a) Monocytes were gated on their typical FSC/SSC appearance and on the presence of CD14. b) Representative histogram showed the level of CD1d expression on monocytes at different time points, day -1 (black line), day 0 (gray line), 2 week (dotted line) in comparison to isotype control (shaded gray) in DF and DHF groups and in healthy control. The level of CD1d expression was expressed as the difference in mean fluorescence intensity (dMFI) between CD1d staining and isotype control of each sample. c) Each dot represents CD1d dMFI on monocytes of each patient in DF and DHF groups at 3 different time points, the line represents median of each group. d) Monocytes CD1d dMFI during febrile phase (day -1) (left) and 2 weeks (right) of healthy, OFI, DF and DHF. e) Monocytes CD1d dMFI during the course of dengue infection. Each line connects data from each patient at different time points. Mann- Whitney test (c–d), and Wilcoxon signed rank test (e) were used for statistical comparison, p<0.05 was considered as statistically significant difference (*p<0.05, **p<0.01).

Our next question was how iNKT cells get activated during acute DV infection. iNKT cells were known to be activated by cognate recognition of iNKT cell receptor to antigen presented on CD1d. In some circumstances, they can be activated indirectly by cytokines or might require both cognate recognition and cytokine-driven activation [54]. To first investigate if iNKT cells could be activated through the CD1d-dependent pathway, we measured CD1d expression on monocytes from the same patient samples used to study iNKT cells. Monocytes were examined because they are abundant antigen presenting cells in peripheral blood, known to be infected by dengue virus both *in vivo* and *in vitro*, and also known to activate iNKT cells in other circumstances. Monocytes were first gated (Figure 4a) and the level of CD1d expression on monocytes at different time points was then analyzed in comparison to isotype control in both DF and DHF groups (Figure 4b). Interestingly, the expression of CD1d was highest on monocytes during day -1 and day 0, in both DF (median DMFI 16058;13304–18998 (day -1) and 15682; 13820–20191 (day 0)) and DHF (17499; 14987–22505 (day -1) and 17991; 15896–21235 (day 0)) patients. The level of CD1d expression decreased significantly by 2 weeks after fever subsided in both DF (10184; 7962–11852) (p = 0.002 (day -1 vs. 2 weeks), p = 0.01 (day 0 vs. 2 weeks)) and DHF (9645; 8999–12712) (p = 0.008 (day -1 vs. 2 weeks), p = 0.002 (day 0 vs. 2 weeks)) (Figure 4c) to the same level as healthy control (10504; 9704–13541) (Figure 4d). The similar trend is clearly observed when data from the same patients at 3 different time points were analyzed (Figure 4e). Monocytic CD1d expression at 6 months was similar to those at 2 weeks (data not shown). Furthermore, when comparing between patients with different dengue disease severity, the level of monocyte CD1d expression at day -1 was higher in DHF than in healthy control (10504; 9704–13541) (p = 0.01), but not significantly higher than that of DF (p = 0.27) (Figure 4d). Thus, CD1d expression on monocytes was upregulated in acute DV infection. Moreover, the level of CD1d expression on monocytes positively correlates, although weakly, with the level of iNKT cell activation (r = 0.35, p<0.05). The correlation is stronger when the DHF subgroup was analyzed (r  =  0.7, p = 0.002) (Figure S5). These results suggested that CD1d was upregulated in monocytes during acute DV infection. However, future experiments are needed to delineate if iNKT cells were actually activated in a CD1d-dependent manner in DV infection.

### Cytotoxic T cells were activated in relation to iNKT cell activation

In general, once iNKT cells get activated, they were known to influence adaptive T cell immune response. To investigate if iNKT cells activation were associated with the activation of conventional T cells in DV infection, the percentage of CD8^+^ and CD4^+^ T cells as well as their activation state were evaluated in the PBMC samples that were used for iNKT cells studies. Lymphocytes were pregated based on their characteristic appearance on forward and side scattered dot plot. CD8^+^ and CD4^+^ conventional T cells were then identified by the expression of CD8 or CD4 together with CD3. CD69 in comparison with isotype control was used as activation marker of both CD4^+^ and CD8^+^ T cells (Figure S6a, c).

**Figure 5:**
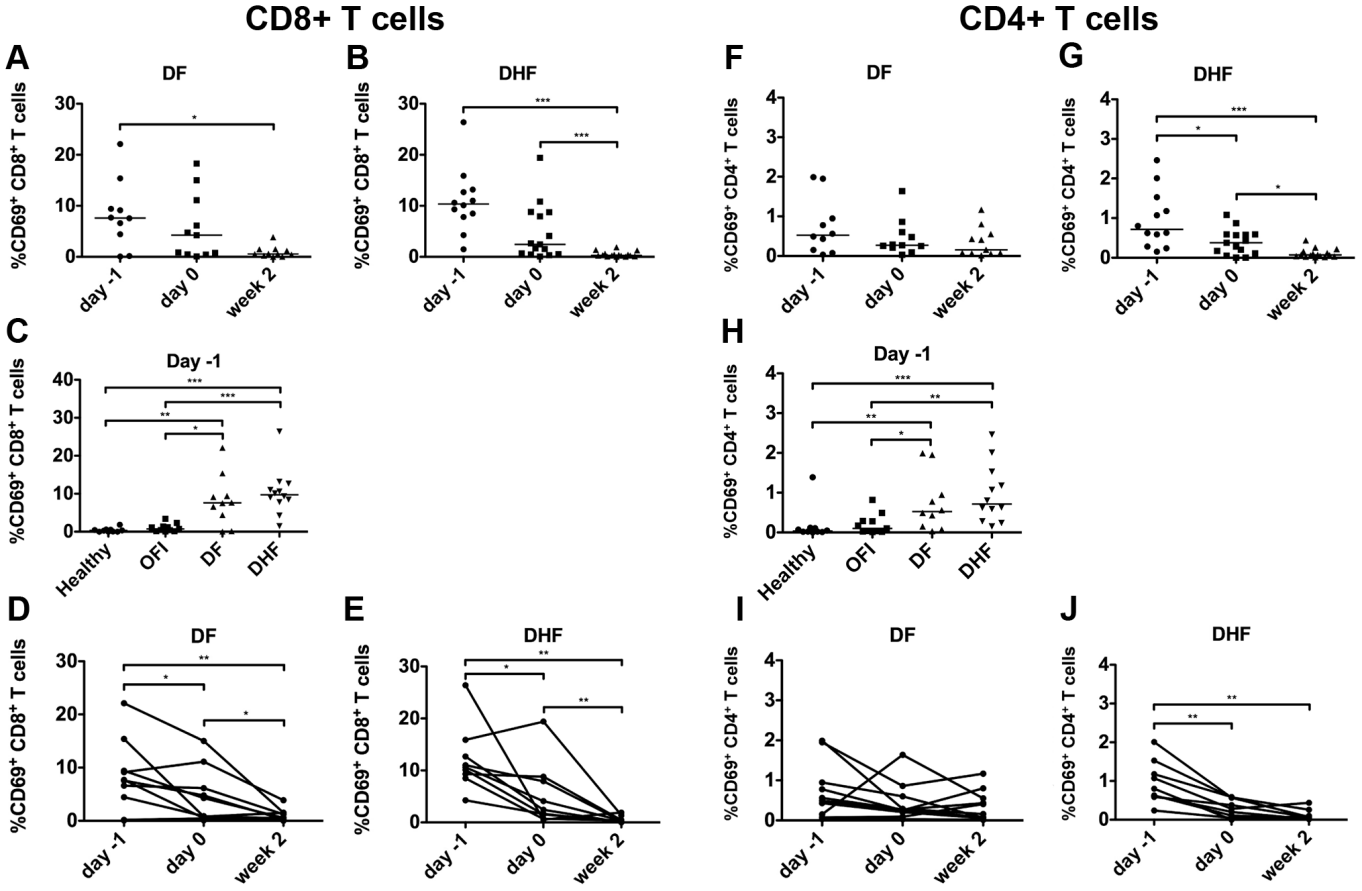
Activation of conventional T cells during acute phase of dengue viral infection. CD8^+^ and CD4^+^ conventional T cells were identified by the expression of CD8 or CD4 together with CD3 within lymphocytes population. The expression of CD69 on CD8^+^ or CD4^+^ conventional was gated in comparison to isotype control. Each dot represents percentage of CD69^+^CD8^+^ (a–e) and CD69^+^CD4^+^ (f–j) conventional T cells of each patients in DF (a, f) and DHF (b, g) group at 3 different time points, the line indicate median of each group. e, h) Dot plot summarized percentage of CD69^+^ CD8^+^ (c) and CD69^+^ CD4^+^ (h) conventional T cells during day -1 of each group of patients in comparison to OFI and healthy controls. Percentage of CD69^+^ CD8^+^ (d, e) and CD69^+^ CD4^+^ (i, j) conventional T cells during the course of dengue infection in DF (d, i) and DHF (e, j), each line connects data from each patient at various time points. Mann-Whitney test (a–c, f–h), and Wilcoxon signed rank test (d–e, i–j) were used for statistical comparison, *p<0.05, **p<0.01, ***p<0.001, p<0.05 was considered as statistically significant difference.

Similar to iNKT cell activation, conventional CD8^+^ T cells were activated during acute phase of dengue infection in both DF and DHF patients (Figure 5a,b). The percentage of CD69^+^CD8^+^ conventional T cells at day -1 (7.62%; 3.36–10.92) was higher than 2 weeks after fever subsided (0.56%; 0.24–1.48) in patients with DF (p = 0.01) (Figure 5a). In patients with DHF, the percentage of CD69^+^ CD8^+^ conventional T cells was higher at day -1 (10.35%; 7.99–13.08) and day 0 (2.40%; 0.52–8.71) when compared to 2 weeks after fever subsided (0.30%; 0.14–0.57) (p<0.0001 and p = 0.0008 respectively) (Figure 5b). Furthermore, during febrile phase, patients with DF (7.62%; 3.36–10.92) and DHF (10.35%; 7.99–13.08) have significantly higher percentage of activated CD8^+^ T cells when compared to OFI (0.76%; 0.15–1.38), and healthy controls (0.32%; 0.026–0.565) (DF vs OFI, p = 0.01, DF vs healthy, p = 0.005, DHF vs OFI, p<0.0001 and DHF vs healthy, p = 0.0001 respectively). At day -1, the percentage of activated CD8^+^ T cells of DHF appeared to be higher than DF, but did not reach statistical significance (Figure 5c). This is different from previous reports that showed a higher CD8 activation in DHF compared to DF [55]–[57], possibly due to the small sample size, high variability of the data and different time point analyzed. When cells from each patient were analyzed at 3 different time points, most of the data showed the percentage of activated CD8^+^ conventional T cells were highest during febrile phase, continuously decreased over the course of infection and almost absent by 2 weeks after fever subsided in both patients with DF (Figure 5d) and DHF (Figure 5e). The activation of CD8^+^ conventional T cells correlates with iNKT cell activation (r = 0.56, p<0.0100) especially in DHF patients (r = 0.69, p<0.0100) (Figure S6b), suggesting that iNKT cell activation may associate with the activation of CD8^+^ conventional T cells especially in DHF patients.

At the analyzed time point, CD4^+^ conventional T cells were also activated during acute DV infection but to a lesser extent than CD8^+^ T cells and were more prominent in DHF patients (Figure 5f–j). In DHF, but not DF patients, CD4^+^T cells showed higher activation during day -1 (0.72%; 0.36–1.44) and day 0 (0.38%; 0.11–0.59) when compared to 2 weeks afterward (0.07%; 0.03–0.15) (p<0.0001, p = 0.013) (Figure 5g, j). During day -1, DF and DHF patients showed increased CD4+ T cells activation when compared to healthy controls or OFI (Figure 5h). No significant difference was observed between DF and DHF (Figure 5h), although it appears that only a few DF patients showed increased CD4^+^ T cell activation (Figure 5h, i). The level of CD4^+^ T cell activation also weakly correlates with the level of iNKT cells activation (r = 0.41, p<0.01), but the correlation is more prominent in DHF (r = 0.57, p<0.01) (Figure S6d).

## Discussion

Our finding is the first to show that human iNKT cells are activated during acute dengue viral infection and the level of activation associates with disease severity. The activation subsided by 2 weeks after fever subsided. Furthermore, the activated iNKT cells from acute DV infected patients produced less IFN-γ in response to subsequent α-GalCer stimulation *ex vivo*. During acute DV infection, monocytes also upregulated CD1d expression and conventional T cells were activated.

The association between the level of iNKT cell activation and the severity of dengue infection in our human data suggests that activated iNKT cells might contribute to the pathogenesis of severe dengue infection. Further investigations are needed to dissect detailed mechanistic involvement of activated iNKT cells in severe dengue disease pathogenesis. It is possible that the activated iNKT cells contribute to immunopathology in severe dengue infection. Alternatively, activated iNKT cells could play a regulatory role that might impede viral clearance resulting in the severe disease. We favor the former hypothesis because our data showed that the activation state of iNKT cells correlates with the activation of conventional T cells known to play pathogenic role in severe disease. In addition, previously published murine data showed that iNKT cell deficient mice suffer less sign of plasma leakage, a cardinal feature of DHF, and survived more than wild type mice [46]. The plasma leakage and death were restored after iNKT cells reconstitution [46]. These data together suggested that iNKT cells might play a detrimental role in dengue virus infection in mice and human.

Despite the finding of iNKT cell activation during acute dengue infection, the change in number of iNKT cell in peripheral blood was not observed during the course of infection. It is possible that iNKT cell migrate from intravascular space to affected tissues unexamined as suggested in some animal models [44]. Moreover, some activated iNKT cells might downregulate their iTCR rendering them undetectable by α-GalCer-loaded CD1d tetramer staining [11].

When the functional capacity of iNKT cells from dengue-infected patients was evaluated, these iNKT cells had reduced α-GalCer mediated IFN-γ production. Six months after fever subsided, their IFN-γ production capacity upon α-GalCer stimulation resumed to the level similarly found in healthy control, possibly from a new pool of iNKT cells. The rate of functional recovery varies among patients as seen at 2 weeks after fever subsided. This finding was not surprising as several previous reports consistently showed an unresponsiveness of previously activated iNKT cells to subsequent stimulation, a phenomenon known as iNKT cell anergy [52], [53], [58]. Similar to our finding, they observed reduction in IFN-γ, but not IL-4, in response to subsequent α-GalCer stimulation. Therefore, this finding support that iNKT cells were activated during acute dengue virus infection *in vivo*, rendering them less responsive to subsequent stimulation *ex vivo*, while their functional capacity returned 6 months after the fever subsided. Interestingly, iNKT cells from acute DF patients has higher IFN-γ/IL-4 ratio after ex-vivo α-GalCer stimulation than those from acute DHF patients. This finding suggests that cytokines pattern produced by iNKT cells could possibly influence DV disease severity. It would be interesting to know the pattern of cytokine production of iNKT cells *in vivo* during the actual dengue infection. Unfortunately, we could detect only small amount of intracellular cytokines on iNKT cells from patients without any stimulation, which may be because they secreted most cytokines *in vivo* or because our detection sensitivity is limited by using cryopreserved PBMC. Further study is warranted to delineate the function of iNKT cells during acute DV infection *in vivo*.

In parallel with iNKT cell activation during acute DV infection, monocytic CD1d surface expression was upregulated. This finding suggests the possible involvement of CD1d on iNKT cell activation through cognate recognition pathway although further experiments are needed to demonstrate how iNKT cells are activated in acute dengue infection. Moreover, CD1d expression on other antigen presenting cells beside monocytes such as dendritic cells may be interesting to examine. Some viruses such as coxakievirus B3 [59] and hepatitis C virus [60] are known to upregulate CD1d expression and activate iNKT cells while others evade iNKT cell recognition by downregulating CD1d, such as Kaposi sarcoma-associated herpes virus [61], herpes simplex virus type 1 [62], HIV type 1 [63], [64] and human papilloma virus [65]. Since viruses do not contain viral lipid antigens, it is postulated that viral infection may alter endogenous lipid presented on CD1d and activate iNKT cells with or without help from cytokines [66], [67]. Since viruses do not contain viral lipid antigens, altered endogenous lipid might be presented on CD1d [29]. Indeed, recent data suggested alteration of lipid metabolism in DV infected cells but their importance in iNKT cell activation is not yet known [68]. Other possible mechanism to activate iNKT cells, especially cytokine mediated activation, was not evaluated and warrants further study. Cytokines of special interest include, but not limited to, IL-12, IL-18, IL-1b and IL-23. Further investigation is needed to delineate the detail of how dengue virus upregulates CD1d and how exactly iNKT cell activation was achieved in human DV infection.

Once iNKT cells are activated, they are known to influence other immune cells both in innate response such as NK cells, dendritic cells and in adaptive T cell and B cell responses [11], [20]. T cells are very important arm of immune defense against viral infection, but T cell overactivation can lead to immunopathology in dengue infection [69], [70]. Our results showed the association between iNKT cells activation, T cell activation and disease severity. Consistently, in murine model, mice without iNKT cells had less T cell response and less dengue disease severity [46]. However, whether iNKT cell activation leads to T cells activation in human DV infection is not known and require further study. The possible crosstalk of iNKT cells with other immune cells such as B cells and NK cells in DV infection are also subjects of interest for future study.

Secondary dengue infection is associated with higher disease severity. It would be interesting to compare iNKT cell response in primary and secondary dengue infection. However, because Thailand is a hyper endemic area, most of our cohort samples are secondary DV infection. Therefore, primary infection was not evaluated in this current study.

In summary, we provide the first evidence that iNKT cells may play a role in human DV infection, one of the most important and expanding global health problems. As neither vaccine nor specific treatment are available for DV infection, and detrimental immune response might cause severe disease rather than protect the host, a better understanding of how immune response are regulated is crucial. We showed here the possible involvement of iNKT cells in human DV infection. Therefore, CD1d and iNKT cells may serve as attractive targets for designing novel strategy to help alleviate suffering from DV infection in the future.

## Supporting Information

Figure S1 **The percentage and absolute number of peripheral blood iNKT cells during the course of dengue infection.** a, b) The percentage of iNKT cells during the course of dengue infection in each patient with DF (a) and DHF (b). Each line connects data from each patient at different time points. c, d) Each dot represents the absolute numbers of iNKT cells (x10^3^ cells/ml) of each patient in DF (c) and DHF (d) groups at 3 different time points, each line represents median of each group. e) The absolute number of iNKT cells during day -1 of OFI, DF and DHF. f, g) The absolute iNKT cells during the course of dengue infection in DF (f) and DHF (g) groups. Each line connects data from each patient at different time points. Mann-Whitney test (c–e), and Wilcoxon signed rank test (a–b, f–g) were used for statistical comparison, *p<0.0500, **p<0.0100, ***p<0.0010.
(TIF)

Figure S2 **Contour plot of cytokines production by iNKT cells at day 0 and month 6.** Pregated on iNKT cells, representative contour plots show the production of IFN-γ (a) or IL-4 (b) within iNKT cells in comparison to isotype control in healthy, OFI, DF and DHF groups, at day 0 or 6 months, with (stimulated) and without (unstimulated) α-GalCer stimulation.
(TIF)

Figure S3 **Contour plot of cytokines production by iNKT cells at day -1 and week 2.** Pregated on iNKT cells, representative contour plots show the production of IFN-γ (a) or IL-4 (b) within iNKT cells in comparison to isotype control in healthy, OFI, DF and DHF groups, at day 0 or 6 months,with (stimulated) and without (unstimulated) α-GalCer stimulation.
(TIF)

Figure S4 **Cytokines production by iNKT cells from dengue infected patients at day -1 and week2 with and without stimulation with α-GalCer.** Each dot represents percentage of interferon gamma (IFN-γ)^+^ (a-b) and IL-4^+^ (e–f) iNKT cells from DF (a,e) and DHF (b,f) at day -1 and week 2, with (stimulated) and without (unstimulated) α-GalCer stimulation. At day -1 (c, g, i) and week 2 (d, h, j), percentage of IFN-γ^+^ iNKT cells (c,d), IL-4^+^ iNKT cells (g, h) and IFN-γ/IL-4 ratio (i, j) upon α-GalCer stimulation, comparing cells from healthy, OFI, DF and DHF groups. Mann-Whitney test, were used for statistical comparison, p<0.05 was considered as statistically significant difference (*p<0.05).
(TIF)

Figure S5 **CD1d expression on monocytes correlates with the activation of iNKT cells.** Spearman rho's correlation analysis of %CD69^+^ iNKT cells and difference in mean fluorescence intensity (dMFI) of CD1d on monocytes in all patients combined (a) or in only patients with dengue hemorrhagic fever (DHF) (b).
(TIF)

Figure S6 **Activation of conventional T cells correlates with the activation of iNKT cells.** a, c) Representative histogram showed expression of CD69 on CD8^+^ (a) and CD4^+^ (c) conventional T cells in healthy, OFI, DF, and DHF at day -1, day 0 and 2 weeks. Representative histograms show the expression of CD69 (black line) in comparison to isotype control (gray shade). b, d) Spearman rho's correlation analysis of %CD69^+^ iNKT cells and of %CD69^+^CD8^+^T cells in all patients combined (a) or in only patients with dengue hemorrhagic fever (DHF) (b). Spearman rho's correlation analysis of %CD69^+^iNKT cells and of %CD69^+^CD8^+^T cells (b) and %CD69^+^iNKT cells and of %CD69^+^CD4^+^T cells (d) in all patients combined (ALL) or in only patients with dengue hemorrhagic fever (DHF) (d).
(TIF)

Table S1 **Characteristics of the patients (samples used for phenotypic analysis of peripheral blood iNKT cells).**
(PDF)

Table S2 **Characteristics of the patients (samples were used for** ***ex vivo*** **functional analysis of peripheral blood iNKT cells).**
(PDF)
