# Supplementary figures and images for: Invariant NKT Cell Response to Dengue Virus Infection in Human
Source: PLoS Negl Trop Dis. 2014 Jun 19;8(6):e2955. doi: 10.1371/journal.pntd.0002955 (PMC4063705; doi:10.1371/journal.pntd.0002955)

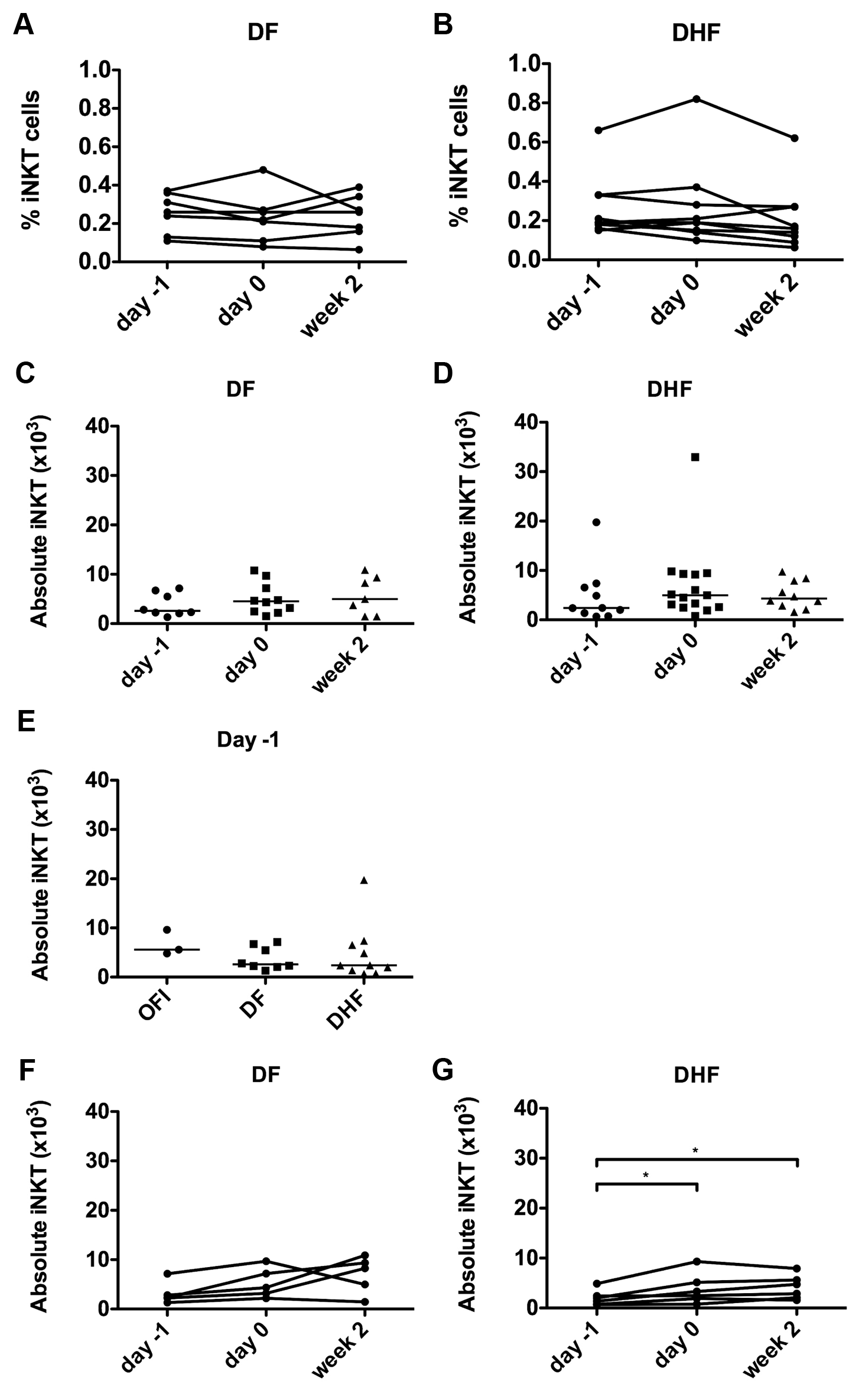

Supplement: Figure S1 — The percentage and absolute number of peripheral blood iNKT cells during the course of dengue infection. a, b) The percentage of iNKT cells during the course of dengue infection in each patient with DF (a) and DHF (b). Each line connects data from each patient at different time points. c, d) Each dot represents the absolute numbers of iNKT cells (x103 cells/ml) of each patient in DF (c) and DHF (d) groups at 3 different time points, each line represents median of each group. e) The absolute number of iNKT cells during day -1 of OFI, DF and DHF. f, g) The absolute iNKT cells during the course of dengue infection in DF (f) and DHF (g) groups. Each line connects data from each patient at different time points. Mann-Whitney test (c–e), and Wilcoxon signed rank test (a–b, f–g) were used for statistical comparison, *p<0.0500, **p<0.0100, ***p<0.0010. (TIF) [file pntd.0002955.s001.tif]

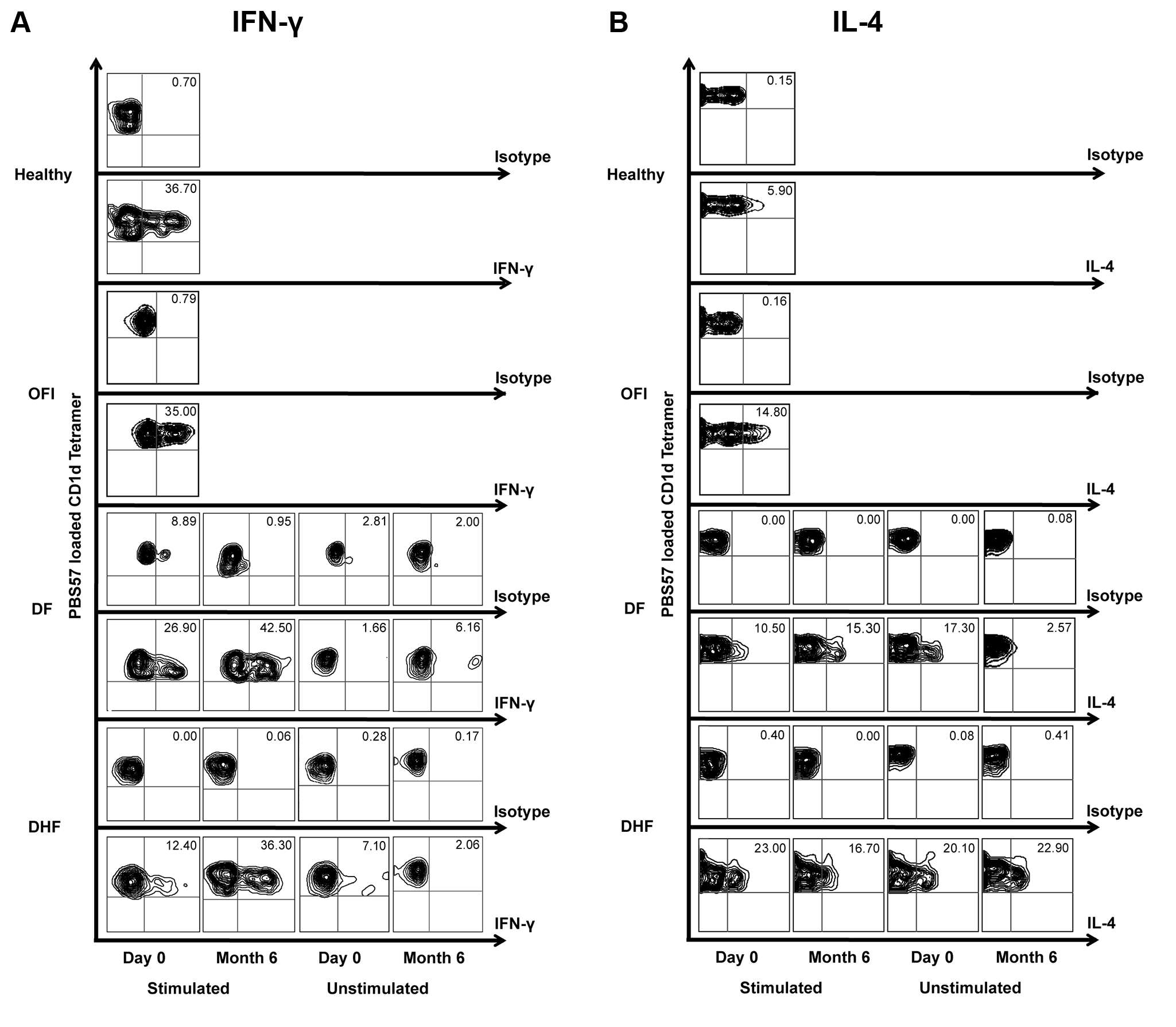

Supplement: Figure S2 — Contour plot of cytokines production by iNKT cells at day 0 and month 6. Pregated on iNKT cells, representative contour plots show the production of IFN-γ (a) or IL-4 (b) within iNKT cells in comparison to isotype control in healthy, OFI, DF and DHF groups, at day 0 or 6 months, with (stimulated) and without (unstimulated) α-GalCer stimulation. (TIF) [file pntd.0002955.s002.tif]

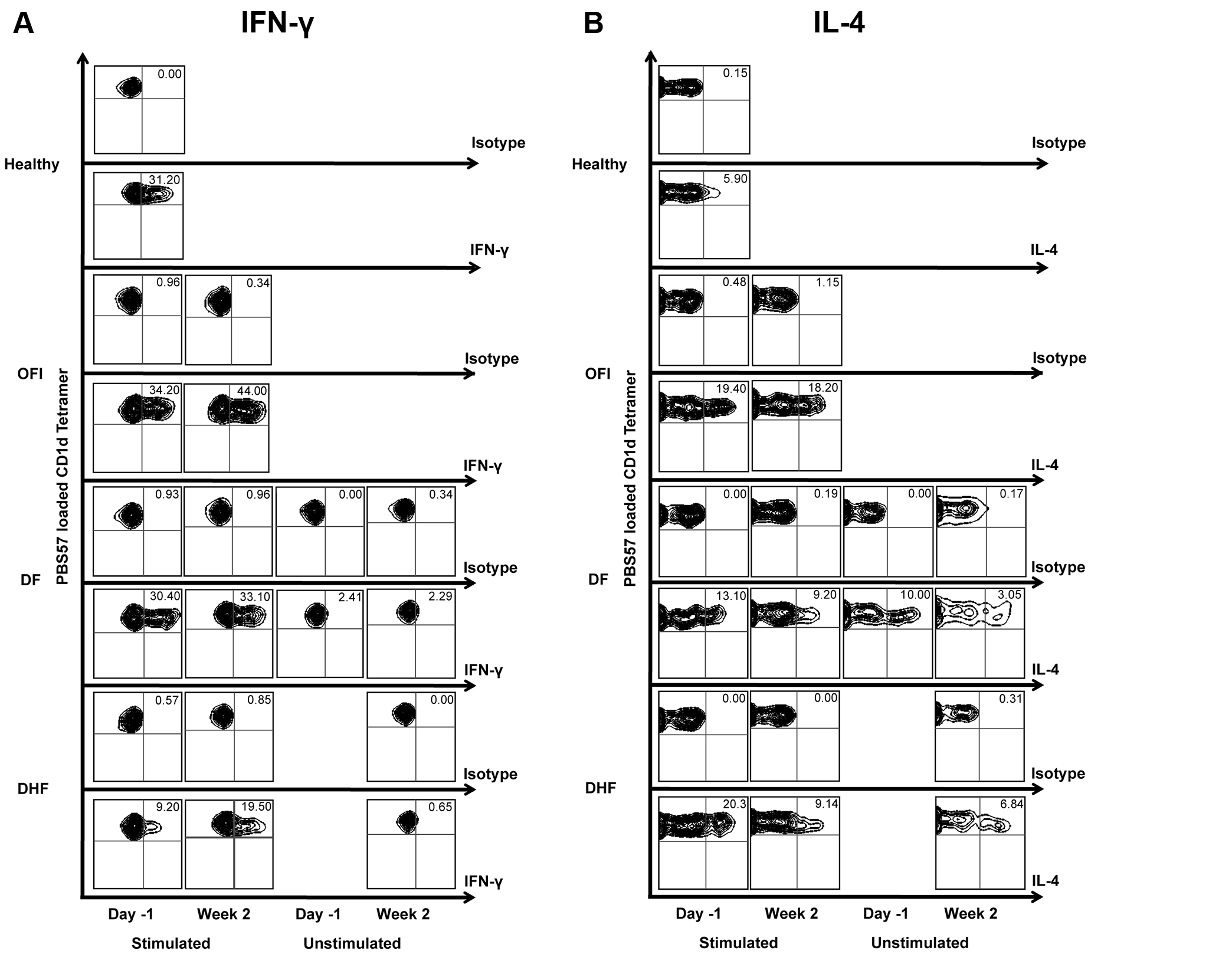

Supplement: Figure S3 — Contour plot of cytokines production by iNKT cells at day -1 and week 2. Pregated on iNKT cells, representative contour plots show the production of IFN-γ (a) or IL-4 (b) within iNKT cells in comparison to isotype control in healthy, OFI, DF and DHF groups, at day 0 or 6 months,with (stimulated) and without (unstimulated) α-GalCer stimulation. (TIF) [file pntd.0002955.s003.tif]

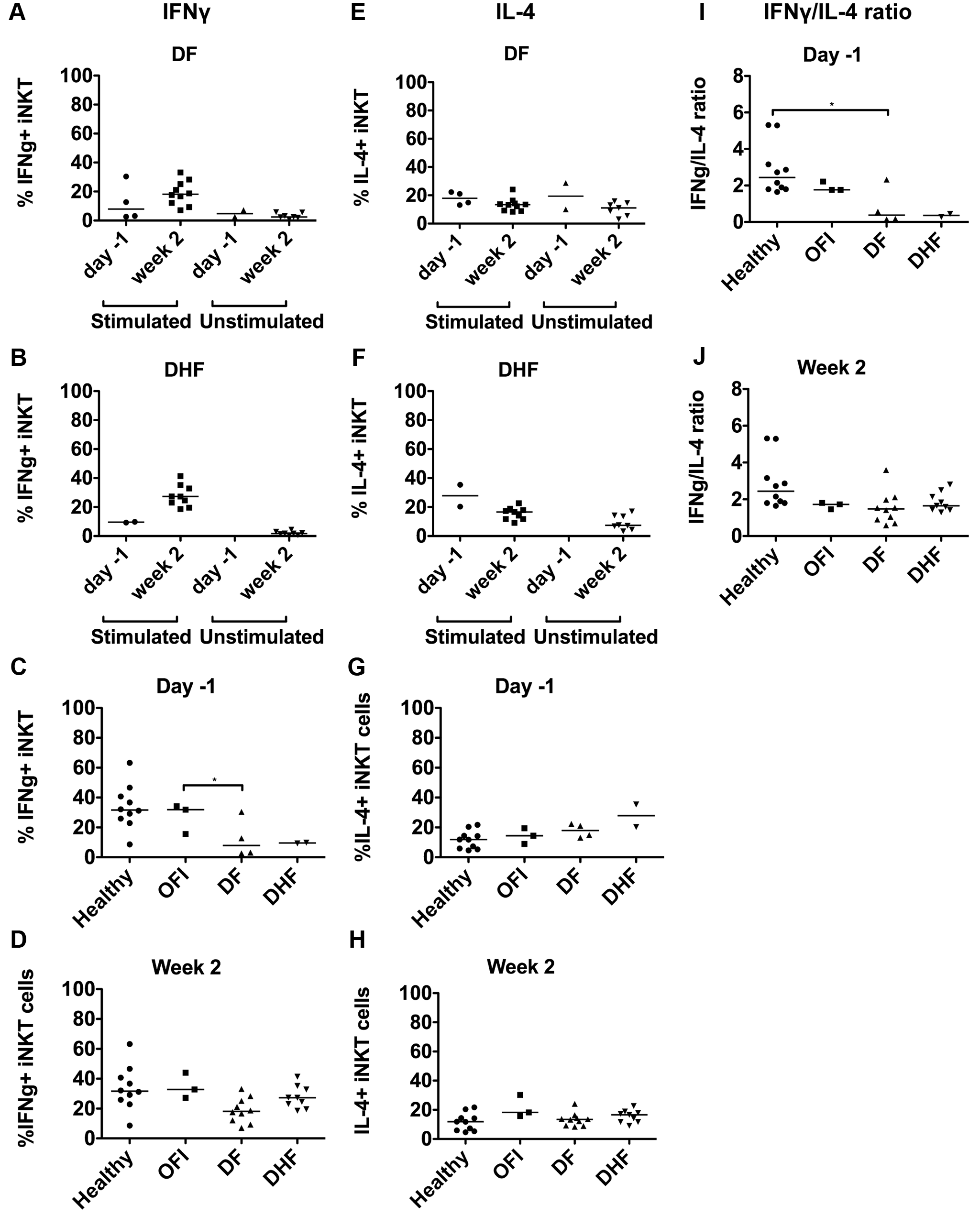

Supplement: Figure S4 — Cytokines production by iNKT cells from dengue infected patients at day -1 and week2 with and without stimulation with α-GalCer. Each dot represents percentage of interferon gamma (IFN-γ)+ (a-b) and IL-4+ (e–f) iNKT cells from DF (a,e) and DHF (b,f) at day -1 and week 2, with (stimulated) and without (unstimulated) α-GalCer stimulation. At day -1 (c, g, i) and week 2 (d, h, j), percentage of IFN-γ+ iNKT cells (c,d), IL-4+ iNKT cells (g, h) and IFN-γ/IL-4 ratio (i, j) upon α-GalCer stimulation, comparing cells from healthy, OFI, DF and DHF groups. Mann-Whitney test, were used for statistical comparison, p<0.05 was considered as statistically significant difference (*p<0.05). (TIF) [file pntd.0002955.s004.tif]

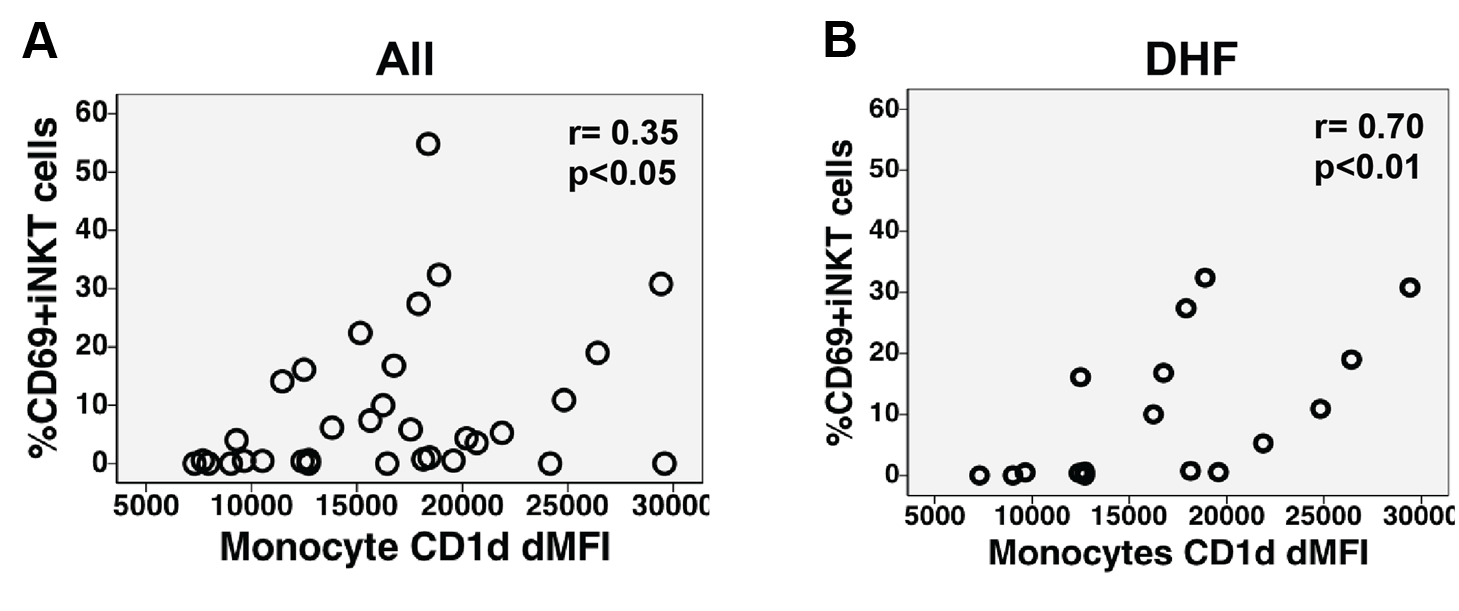

Supplement: Figure S5 — CD1d expression on monocytes correlates with the activation of iNKT cells. Spearman rho's correlation analysis of %CD69+ iNKT cells and difference in mean fluorescence intensity (dMFI) of CD1d on monocytes in all patients combined (a) or in only patients with dengue hemorrhagic fever (DHF) (b). (TIF) [file pntd.0002955.s005.tif]

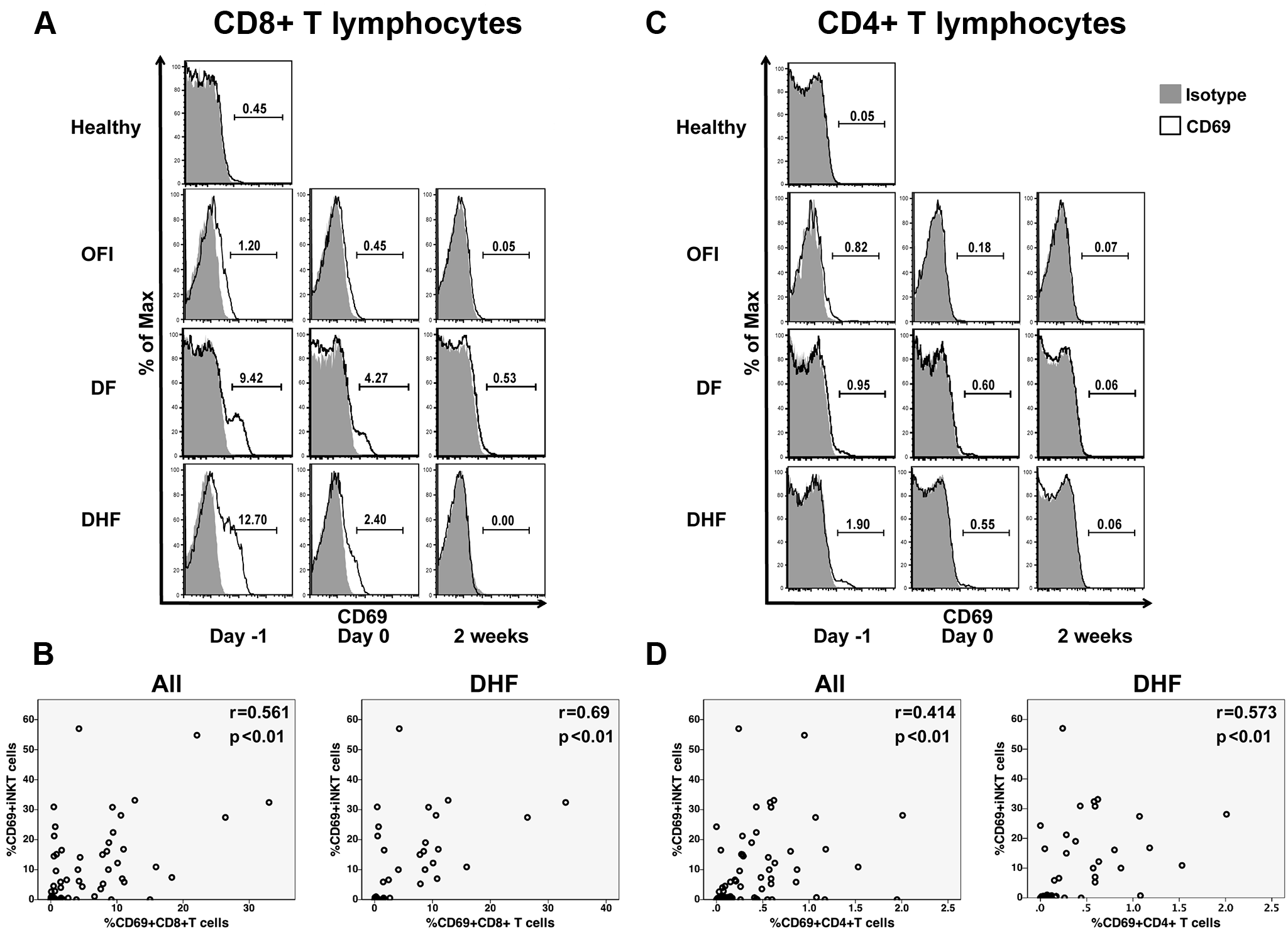

Supplement: Figure S6 — Activation of conventional T cells correlates with the activation of iNKT cells. a, c) Representative histogram showed expression of CD69 on CD8+ (a) and CD4+ (c) conventional T cells in healthy, OFI, DF, and DHF at day -1, day 0 and 2 weeks. Representative histograms show the expression of CD69 (black line) in comparison to isotype control (gray shade). b, d) Spearman rho's correlation analysis of %CD69+ iNKT cells and of %CD69+CD8+T cells in all patients combined (a) or in only patients with dengue hemorrhagic fever (DHF) (b). Spearman rho's correlation analysis of %CD69+iNKT cells and of %CD69+CD8+T cells (b) and %CD69+iNKT cells and of %CD69+CD4+T cells (d) in all patients combined (ALL) or in only patients with dengue hemorrhagic fever (DHF) (d). (TIF) [file pntd.0002955.s006.tif]
