# Supplementary material for: Invariant NKT Cell Response to Dengue Virus Infection in Human
Source: PLoS Negl Trop Dis. 2014 Jun 19;8(6):e2955. doi: 10.1371/journal.pntd.0002955 (PMC4063705; doi:10.1371/journal.pntd.0002955)
Supplement: Table S1 — Characteristics of the patients (samples used for phenotypic analysis of peripheral blood iNKT cells). (PDF) [file pntd.0002955.s007.pdf]

Table S1 Characteristics of the patients (samples used for phenotypic analysis of peripheral blood iNKT cells)

Patients	Age	Gender	Serotype	WBC[A]	Hct[B]	Platelet[C]	AST[D]	ALT[E]	Albumin[F]
DF1	10	F	DV2	4.0	39.0	65.0	42.0	40.0	3.1
DF2	7	F	DV1	2.2	37.0	86.0	26.0	28.0	4.0
DF3	15	M	DV2	2.7	42.7	78.0	26.0	29.0	4.0
DF4	13	M	DV2	2.1	37.3	77.0	73.0	54.0	3.3
DF5	11	F	DV3	2.1	36.0	77.0	67.0	34.0	6.7
DF6	9	F	DV3	2.3	35.0	87.0	107.0	59.0	7.1
DF7	14	M	DV2	3.6	39.0	55.0	96.0	39.0	3.7
DF8	9	M	DV1	3.2	40.0	120.0	30.0	30.0	3.3
DF9	9	M	DV3	1.9	38.9	80.0	118.0	137.0	2.7
DF10	9	F	DV1	2.1	37.4	59.0	118.0	137.0	3.0
DF11	14	F	DV1	1.1	36.5	66.0	64.0	48.0	3.1
**Average**	11	M5:F6	4:4:3:0	2.5	38.1	77.3	69.7	57.7	4.0
**SD**	2.6			0.8	2.1	17.6	36.0	40.5	1.5
DHF1	11	F	DV4	2.6	38.0	23.0	164.0	71.0	3.0
DHF2	8	F	DV4	1.9	36.0	14.0	125.0	57.0	2.2
DHF3	13	F	DV4	2.7	41.0	38.0	452.0	198.0	2.6
DHF4	8	M	DV1	1.8	38.0	142.0	76.0	47.0	3.8
DHF5	12	F	DV3	1.9	41.0	12.0	182.0	43.0	2.7
DHF6	12	M	DV3	1.7	33.5	36.0	1119.0	363.0	2.3
DHF7	11	M	DV2	2.5	40.5	50.0	150.0	93.0	3.4
DHF8	10	F	DV3	3.9	44.0	31.0	288.0	102.0	2.5
DHF9	8	M	DV2	2.6	42.0	63.0	83.0	40.0	3.3
DHF10	13	M	DV2	4.1	34.0	25.0	671.0	274.0	2.9
DHF11	15	M	DV2	2.2	48.9	14.0	113.0	59.0	3.2
DHF12	12	F	DV1	1.9	41.0	12.0	182.0	43.0	2.7
DHF13	9	M	DV3	3	40.0	17.0	409.0	284.0	2.6
DHF14	14	F	DV1	2.6	38.0	60.0	157.0	61.0	2.9
DHF15	10	F	DV1	2.6	44.0	41.0	105.0	68.0	2.2
DHF16	7	F	DV2	1.9	41.0	24.0	101.0	35.0	2.6
DHF17	7	F	DV3	2.8	40.0	25.0	131.0	55.0	2.4
DHF18	10	F	DV1	2.2	42.0	9.0	106.0	30.0	3.0
DHF19	13	F	DV1	1.2	36.4	98.0	75.0	60.0	3.5
**Average**	11	M7:F12	6:5:5:3	2.4	40.0	38.6	246.8	104.4	2.8
**SD**	2.4			0.7	3.7	33.6	262.1	98.6	0.5
**P-value (DF vs DHF)**				0.8459	0.0973	***0.0007	***0.0005	*0.0408	**0.0026

[A]lowest white blood cell count, [B]highest hematocrit, [C]lowest platelet, [D]highest serum aspartate transaminase, [E]highest serum alanine transaminase, [F]lowest serum albumin
