# Supplementary material for: Invariant NKT Cell Response to Dengue Virus Infection in Human
Source: PLoS Negl Trop Dis. 2014 Jun 19;8(6):e2955. doi: 10.1371/journal.pntd.0002955 (PMC4063705; doi:10.1371/journal.pntd.0002955)
Supplement: Table S2 — Characteristics of the patients (samples were used for ex vivo functional analysis of peripheral blood iNKT cells). (PDF) [file pntd.0002955.s008.pdf]

Table S2 Characteristics of the patients (samples were used for *ex vivo* functional analysis of peripheral blood iNKT cells)

**Patients**	**Age**	**Gender**	**Serotype**	**WBC[A]**	**Hct[B]**	**Platelet[C]**	**AST[D]**	**ALT[E]**	**Albumin[F]**
DF3	15	M	DV2	2.7	42.7	78.0	26.0	29.0	4.0
DF6	9	F	DV3	2.3	35.0	87.0	107.0	59.0	7.1
DF7	14	M	DV2	3.6	39.0	55.0	96.0	39.0	3.7
DF12	14	M	DV3	1.5	43.6	25.0	169.0	76.0	2.7
DF13	10	M	DV1	3.8	37.0	116.0	92.0	80.0	3.2
DF14	11	M	DV1	2.1	39.0	54.0	162.0	116.0	2.7
DF15		M	DV2	4.0	38.0	84.0	96.0	71.0	3.9
DF16	11	M	DV1	6.0	38.0	123.0	95.0	63.0	3.2
DF17	13	M	DV3	3.7	41.8	119.0	32.0	24.0	3.6
DF18	9	F	DV1	2.0	39.0	51.0	178.0	73.0	3.3
DF19	11	F	DV1	3.8	37.0	134.0	66.0	43.0	3.5
DF20	11	F	DV1	2.9	39.0	61.0	68.0	42.0	3.3
DF21	14	F	DV1	2.5	36.0	71.0	475.0	259.0	3.4
DF22	13	F	DV1	3.0	40.0	55.0	194.0	89.0	2.8
**Average**	12	M8:F6	8:3:3:0	3.1	38.9	79.5	132.6	75.9	3.6
**SD**	1.9			1.1	2.5	32.7	111.5	58.4	1.1
DHF6	12	M	DV3	1.7	33.5	36.0	1119.0	363.0	2.3
DHF8	10	F	DV3	3.9	44.0	31.0	288.0	102.0	2.5
DHF10	13	M	DV2	4.1	34.0	25.0	671.0	274.0	2.9
DHF11	15	M	DV2	2.2	48.9	14.0	113.0	59.0	3.2
DHF14	14	F	DV1	2.6	38.0	60.0	157.0	61.0	2.9
DHF15	10	F	DV1	2.6	44.0	41.0	105.0	68.0	2.2
DHF17	7	F	DV3	2.8	40.0	25.0	131.0	55.0	2.4
DHF20	12	M	DV2	1.4	39.8	16.0	201.0	123.0	1.6
DHF21	13	M	DV2	2.4	46.6	14.0	205.0	136.0	2.4
DHF22	2	M	DV1	1.9	38.0	8.0	130.0	52.0	4.0
DHF23	13	F	DV1	2.1	47.0	22.0	137.0	67.0	2.9
DHF24	12	M	DV1	4.1	33.0	51.0	137.0	28.0	3.3
**Average**	11	M7:F5	5:4:3:0	2.7	40.6	28.6	282.8	115.7	2.7
**SD**	3.4			0.9	5.5	15.9	305.7	101.4	0.6
**P-value (DF vs DHF)**				0.3544	0.4088	***0.0002	*0.0221	0.3545	**0.0074

[A]lowest white blood cell count, [B]highest hematocrit, [C]lowest platelet, [D]highest serum aspartate transaminase, [E]highest serum alanine transaminase, [F]lowest serum albumin
